# Species-specific variation in nesting and postfledging resource selection for two forest breeding migrant songbirds

**DOI:** 10.1371/journal.pone.0179524

**Published:** 2017-06-14

**Authors:** Julianna M. A. Jenkins, Frank R. Thompson, John Faaborg

**Affiliations:** 1 School of Natural Resources, University of Missouri, Columbia, Missouri, United States of America; 2 United States Forest Service Northern Research Station, Columbia, Missouri, United States of America; 3 Division of Biological Sciences, University of Missouri, Columbia, Missouri, United States of America; Weyerhaeuser Company, UNITED STATES

## Abstract

Habitat selection is a fundamental component of community ecology, population ecology, and evolutionary biology and can be especially important to species with complex annual habitat requirements, such as migratory birds. Resource preferences on the breeding grounds may change during the postfledging period for migrant songbirds, however, the degree to which selection changes, timing of change, and whether all or only a few species alter their resource use is unclear. We compared resource selection for nest sites and resource selection by postfledging juvenile ovenbirds (*Seiurus aurocapilla*) and Acadian flycatchers (*Empidonax virescens*) followed with radio telemetry in Missouri mature forest fragments from 2012−2015. We used Bayesian discrete choice modeling to evaluate support for local vegetation characteristics on the probability of selection for nest sites and locations utilized by different ages of postfledging juveniles. Patterns of resource selection variation were species-specific. Resource selection models indicated that Acadian flycatcher habitat selection criteria were similar for nesting and dependent postfledging juveniles and selection criteria diverged when juveniles became independent from adults. After independence, flycatcher resource selection was more associated with understory foliage density. Ovenbirds differed in selection criteria between the nesting and postfledging periods. Fledgling ovenbirds selected areas with higher densities of understory structure compared to nest sites, and the effect of foliage density on selection increased as juveniles aged and gained independence. The differences observed between two sympatric forest nesting species, in both the timing and degree of change in resource selection criteria over the course of the breeding season, illustrates the importance of considering species-specific traits and postfledging requirements when developing conservation efforts, especially when foraging guilds or prey bases differ. We recommend that postfledging habitat selection be considered in future conservation efforts dealing with Neotropical migrants and other forest breeding songbirds.

## Introduction

Habitat selection is a fundamental component of community ecology, population ecology and management, and evolutionary biology [1]. The habitat requirements of migratory species, such as Neotropical migrant birds, change over the course of their complex annual cycle [2,3]. Assessments of nesting habitat and breeding densities remain the primary measures used for conservation planning and management for Neotropical migrant birds in North America [4]. However, there is a growing body of evidence that habitat preferences change for many species after juveniles leave the nest [5]. More information is needed on patterns of postfledging habitat use, especially in contrast to nest site characteristics. If the magnitude of change for habitat preference postfledging is great and this change occurs during a period of high juvenile mortality, conservation may need to consider both nesting and postfledging requirements to be successful. Alternatively, if resource selection does not change through the breeding season, management that focuses on nesting habitat may suffice.

Some of the best evidence for shifts in habitat selection during the breeding season come from birds nesting in mature forest. Many forest interior birds commonly nest only in large, mature, open understory stands. However, in the last 20 years, studies have captured postfledging individuals of these species in early successional forest, wildlife openings, riparian forest, and regenerating harvested forest, suggesting large shifts in resource requirements from nesting to postfledging [6–8]. Structurally complex areas with high stem and foliage densities may mitigate risk of predation [9] and offer high food densities [7,10,11]. However, catching a bird in a habitat does not mean that it relies on that habitat exclusively. Relatively few species have been monitored across both nesting and postfledging periods [5] and the majority of postfledging telemetry work has been done with ground or shrub nesting species [5]. Further investigation is needed on the extent and timing of changes to resource selection and whether all or a few species or nesting guilds change their habitat use postfledging [7,12].

Ovenbird (*Seiurus aurocapilla*) and Acadian flycatcher (*Empidonax virescens*) are insectivorous Neotropical migrant songbirds that nest within similar areas of mature deciduous forests in eastern North America. Both species are considered area-dependent breeders, requiring large contiguous interior forested habitat for breeding, and thus are considered sensitive to forest fragmentation [13,14]. Ovenbirds build nests on the ground and spend the majority of their time foraging in leaf litter. Acadian flycatchers build open-cup nests in low-canopy trees and forage by sallying in open areas under closed canopy. For the first several weeks, fledglings remain with family groups in a core natal area, typically near nest sites. Mortality rates are highest early in this dependent postfledging period as they gain mobility and foraging skills [15–17]. After gaining independence from adults several weeks postfledging, longer dispersal movements away from natal areas become common [18]. Postfledging Acadian flycatchers in Ohio riparian forests were found in areas with significantly greater shrub cover than seen at nest sites [17]. Postfledging ovenbirds have been reported using areas of dense cover including nonbreeding habitats such as clear-cuts or secondary growth patches in contiguous forests [7,15,19–21]. These changes in habitat-use suggest that habitat selection differs between the nesting and postfledging periods for both species.

We quantified resource selection by nesting adults (nest site selection) and postfledging juveniles for both ovenbirds and Acadian flycatchers in Missouri forest fragments to examine 1) whether resource selection changes over the breeding season (nesting and postfledging) and 2) if changes in resource selection are related to postfledging development. While adults likely determine much of the habitat use in the dependent postfledging period, we refer to all resource selection out of the nest as ‘postfledge juvenile’ selection for consistency. We used Bayesian discrete choice modeling to evaluate and compare resource selection between breeding season life stages.

## Methods

### Study area

We studied breeding season ecology on three forested sites in Central Missouri (Boone, Randolph, and Howard counties) from 2012−2015. We surveyed the Thomas S. Baskett Wildlife Research and Education Center (38° 44′N, 92°12′W; 890 ha) from 2012−2015, Rudolf Bennitt State Conservation Area (39° 8' N, 92° 15' W; 1146 ha) from 2013−2015, and Three Creeks Conservation Area (38° 49′N, 92°17′W; 575 ha) from 2014−2015. Acadian flycatchers were present at all three sites, while nesting ovenbirds were only present at Rudolf and Baskett forests. In 2012 we only monitored Baskett ovenbirds.

All study sites were mixed-hardwood forest with overstory dominated by oak (*Querus spp*.) and hickory (*Carya spp*.), interspersed with stands of successional red cedar (*Juniperus virginiana*) in a landscape that was approximately 35 percent forest and 65 percent pasture, cropland, and old-fields. The understory plant community included flowering dogwood (*Cornus florida*), viburnum (*Viburnum sp*.), hophornbeam (*Ostrya virginiana*), serviceberry (*Amelanchier arborea*), and sugar maple (*Acer saccharinum*). Ground cover included aromatic sumac (*Rhus aromatica*), Virginia creeper (*Parthenicissus sp*.), buck brush (*Symphoricarpos orbiculatus*), and poison ivy (*Toxicodendron radicans*). Topography consists of ridge tops separated by ravines that feed ephemeral and perennial streams, with narrow floodplains.

### Nest monitoring and radio telemetry

We searched forested areas daily for nests using adult cues and systematic searches from mid-May to early-August and monitored located nests every three to five days following standard methods until nest failure or fledging [22]. Search effort focused on areas of high breeding density, which may reduce the variability of nest selection coefficients [23], but maximized the number of monitored juveniles. We captured all available nestlings on the day of projected fledging (8 days posthatch for ovenbirds and 13 days posthatch for Acadian flycatchers, where day 0 is hatch day). We attached colored-leg bands and a standard U.S. Geological Survey (USGS) leg band to all captured ovenbirds and attached radio transmitters to 1–3 individuals per brood. We applied more than one transmitter to a random subset of ovenbird broods to increase sample size and because ovenbirds split broods between adults postfledging [14]. All captured Acadian flycatcher nestlings received a standard USGS leg band and one juvenile per nest received a single colored leg band and a radio transmitter. Transmitters were attached using a leg-loop harness made with flexible cording [24]. In 2012, transmitters weighed < 5% of ovenbird juvenile mass at time of attachment, and had an expected battery life of 22 days (0.55 g, model A1015 Advanced Telemetry Systems (ATS), Itasca, MN, USA). In 2013–2015, transmitters weighed < 3% of ovenbird and Acadian flycatcher body mass at time of attachment, and had an expected battery life of 44, 29, and 44 days respectively (0.3 g, 2013 & 2015: model A2414 ATS, 2014: model PicoPip Ag337 Biotrack, Wareham, Dorset, UK).

We located juvenile birds daily between 0600–1400 hours, or as close to every day as possible, using handheld receivers (model R410-ATS and Model R1000-Communication Specialists Inc. Orange, CA USA) and handheld directional antennas (Yagi 3-element and H-Type ATS). We located individuals until the transmitter signal was no longer detectable (transmitter battery failure or dispersal out of study area) or until we determined mortality. We recorded locations in Universal Transverse Mercator (UTM) coordinates with handheld GPS units (GPS error < 10 m). We recorded the coordinates of the location where we first sighted or flushed the individual. We tried to limit disruption, but our presence may have affected postfledging habitat use; some birds may have sought cover when researchers approached. The University of Missouri’s Animal Care and Use Committee (protocols # 7463 and # 8418) and the U.S. Federal Bird Banding Lab (permit # 09518—AL) approved the field methods of this study. We received permission to exceed the 3% weight restriction in 2012 by the U.S. Federal Bird Banding Lab as part of a larger pilot study.

### Vegetation structure

We sampled environmental resources at used and 2 random available locations for nests found in 2013–2015 and live juvenile locations in 2012–2015. Each group of 3 locations (1 used & 2 associated random locations) is hereafter referred to as a choice-set of available resources. Random points were 50 m away at a random azimuth from the used location. We considered random sites as available due to their close proximity to use sites; 50 m is within the range of juvenile daily movements for both species throughout the postfledging period [18]. We constrained random samples to ‘reasonable’ habitats based upon prior knowledge of species natural history. For example, we did not allow a random sample for either species to occur in open water and we allowed random samples for Acadian flycatcher juveniles but not ovenbird juveniles to occur over a canopy covered road. If a random location was inappropriate, we chose a new random azimuth until we encountered a suitable location. We were not able to sample choice-sets for all juvenile locations due to the time intensive nature of sampling. Instead, we sampled choice-sets for every other juvenile relocation and increased our sampling to every observation (once per day) of random individuals when workload allowed.

We calculated canopy cover at each point using the average of four spherical densiometer readings (one in each cardinal direction). We averaged litter depth measurements taken at the central point and 2 m from the central point in each cardinal direction. We measured the diameter at breast height (DBH), of all stems greater than 3 cm DBH in a 10-factor basal area wedge plot. We calculated stem densities per hectare of saplings (3.0−12.5 cm DBH), pole timber (12.5−27.5 cm DBH), and saw timber (> 27.5 cm DBH [25]). We estimated understory foliage density using the average of four density board (0.3 m x 2 m) measurements taken from 11.3 m in each cardinal direction from the central point. We calculated distance to nearest nonforest edge for points remotely in ArcGIS [26]. Nonforest edge included all forest boundaries adjacent to ponds, roads, and powerline cuts, and other landcover classes that were visible from aerial photos (http://msdis.missouri.edu/; USDA-FSA Aerial Photography Field Office); we did not consider trails and roads with full canopy coverage edge.

### Resource selection models

We used multinomial logit discrete choice models in a Bayesian framework to model the probability an individual nester or postfledging individual would select a location if given a choice between 3 locations available at the time (i.e. choice-set [27,28]). Unlike other resource selection analysis of used vs. available locations, such as logistic regression, discrete choice models rely upon comparisons within choice-sets rather than comparisons across all locations over time. The composition of choice-sets are allowed to change over time and between individuals, so there is no variation in factors that often influence selection, such as an individual’s age or habitat availability [29]. We modeled the utility of each used or random available location within each choice-set as a linear function of vegetation characteristics (litter depth + understory foliage density + sapling density + pole timber density + saw timber density + canopy cover), their interaction with distance to edge, and regression coefficients. We calculated the relative probability of use as a function of those utilities (e.g. [30]). We utilized a hierarchical Bayesian framework to account for repeated observations of fledglings and modeled population-level resource selection for fledglings by assuming that individual-level coefficients arose from normal population-level distributions [28,31]. Since all parameters in Bayesian analysis are treated as random variables with probability distributions, both the population average and variance can be examined fairly simply and individuals with fewer observations can be incorporated and will tend to conform more strongly to the population distribution [28]. For ease of reading, we hereafter refer to each regression coefficient distribution by the name of the associated vegetation covariate. For each covariate coefficient, we present the mean of the posterior distribution (β), the 95% credible interval (CRI), and the proportion of the posterior distribution with the same sign as the mean (*f)*. Higher values of *f* (approaching 1) represent increasing confidence in the direction of the covariate effect. When variables did not have strong evidence of interactive effects with distance to edge (*f* < 0.90), we calculated selection ratios from model coefficients (selection ratio = exp [β]). The selection ratio measures the multiplicative change in probability of selection when a covariate changed by one unit, assuming all others remained constant [29]. We did not calculate selection ratios when interactions were strongly supported, since selection depended upon more than one coefficient. We estimated and interpreted relative probability of use curves (± 95% CRI) over the observed range of covariates of interest, while holding other covariates at their mean [29]; when edge interaction effects were present, curves were created for both the 10th percentile and 90th percentile edge distances.

We hypothesized that resource selection preferences would not be consistent throughout the postfledging period, but would change as fledglings developed. We categorized postfledging period choice-sets into three stages based upon developmental milestones and published mortally risk periods. The highest postfledging mortality rates for both species are reported in the first week out of the nest when fledglings are unable to make long flights and depend on adults for food [5,32,33]. After week one, mortality rates rapidly decline and juveniles increase their daily movements and foraging effort but remain in family groups near natal territories for several weeks [5,18]. After independence, juveniles no longer receive supplemental food and many disperse from natal areas [34]. We modeled separate population-level parameter distributions for early dependent fledglings (first week out of nest, cared for by parents), late dependent fledgling (fledglings > 8 days post-fledge and cared for by parents), and independent fledglings (no parental care).

For all models, posterior distributions for each parameter were estimated using Markov chain Monte Carlo (MCMC) methods implemented in JAGS [35] using the jagsUI package [36] in program R [37]. In cases where we followed more than one ovenbird per brood, we used data from one individual to decrease variation due to brood effects and other correlations among siblings. We selected vague prior distributions for all model parameters. We assumed normal *N*(0, 0.01) prior distributions on all nest regression coefficients and all juvenile population-level mean hyperparameters. We assumed diffuse inverse-gamma distributions *Ɣ*(1, 0.0001) for each juvenile standard deviation hyperparameter. We ran 3 chains for each model, using trace plots to determine an adequate burn-in phase and we ran chains until the Brooks-Gelman-Rubin convergence diagnostic suggested adequate convergence (Rhat ≤ 1.1 for all monitored parameters [38]) Models differed in complexity and required between 20000 and 250000 post-burn iterations to achieve convergence. We calculated Estrella’s R^2^ as an indication of model fit for our discrete choice models (e.g., [30,39]. Values of Estrella’s R^2^ range from 0 (predicts at random) to 1 (perfect fit), with intermediate values of 0.25 and 0.50 generally considered to indicate modest, and strong predictive accuracy [39].

## Results

### Acadian flycatchers

We monitored 264 Acadian flycatcher nests and attached radio transmitters to 45 Acadian flycatchers from 45 broods from 2013–2015. We relocated fledglings every 1.59 ± 0.06 days for a maximum of 45 days postfledging [18,32]. We collected 170 complete choice-sets for Acadian flycatcher nests and 422 total choice-sets for 39 Acadian flycatcher juveniles with 10.8 ± 0.97 (range 1−20) sets per individual (138 sets from 39 early dependent individuals, 172 choice-sets from 30 late dependent individuals, and 112 choice-sets from 24 independent fledglings; S1 Table). Nest and postfledging selection models performed better than the null model of random selection; however, Estrella’s R^2^ indicated both Acadian flycatcher nest and juvenile selection models had low predictive accuracy overall, 0.18 and 0.16, respectively.

**Fig 1:**
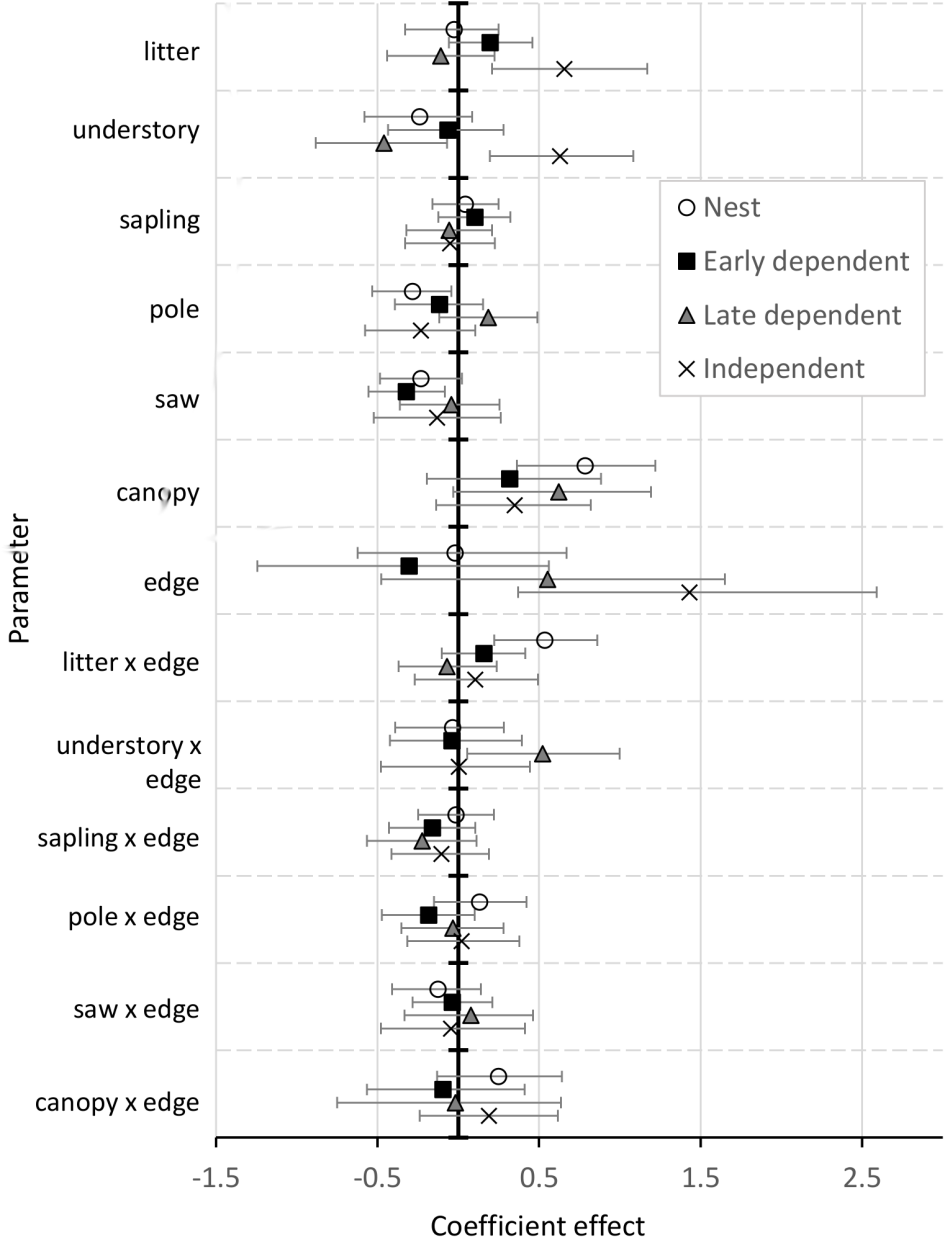
Parameter coefficients from Acadian flycatcher discrete choice models. Mean population-level parameter coefficients and 95% credible intervals from Acadian flycatcher discrete choice models for nests, dependent fledglings in the first week out of the nest (early dependent), dependent fledglings after the first week out of the nest (late dependent), and independent fledglings (> 19 days post-fledge) in Missouri from 2013−2015.

**Fig 2:**
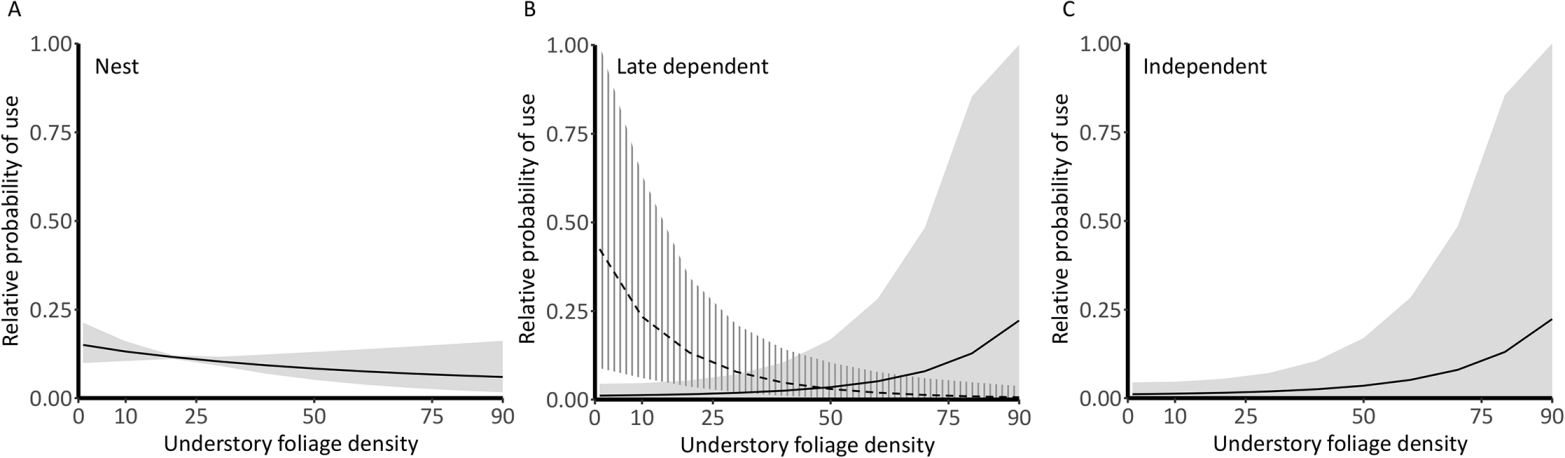
Understory foliage density affected location utility differently across Acadian flycatcher life-stages. The estimated relative probability of use (± 95% credible intervals) for Acadian flycatchers as a function of understory foliage density for nest sites (A), late dependent fledglings near (dotted lines) and far (solid lines) from nonforest edge (B), and for independent fledglings (C) in Missouri, 2013−2015. Probability curves were created for each stage by holding non-focal covariates at their means; when edge interaction effects were present, curves were created using the 10^th^ percentile (near edge = 20 m) and 90^th^ percentiles (far from edge = 300 m).

Selection coefficient CRIs overlapped for nesting, early dependent, and late dependent stages, suggesting relatively consistent resource selection until juvenile independence from adults when coefficient CRIs for litter depth, understory density, and distance to edge diverged from earlier categories (Fig 1, S2 Table). Across all flycatcher nesting and postfledging stages, there was support for a positive effect of canopy cover on resource use (*f* ≥ 0.88, Fig 1), with mean selection ratios ranging from 1.37 (95% CRI 0.82 to 2.42) in the early dependent period to 2.19 (95% CRI 1.44 to 3.39) for nest site selection. Understory foliage density affected location utility in different ways across flycatcher life-stages (Fig 2). During nest site selection, understory foliage density was negatively associated with utility (*f* = 0.92) with a selection ratio of 0.79 (95% CRI 0.62 to 1.02, Fig 2A). During the late dependent period, the effect of understory density interacted with distance to edge (*f* = 0.98); a late dependent postfledging flycatcher near nonforest edge (~20 m from edge) was more likely to utilize a location with lower understory foliage density, while an individual in the core forest (~300 m from edge) was more likely to select a location with higher understory density (Fig 2B). After independence, understory density was positively associated with site selection (*f* = 1) with a selection ratio of 1.88 (95% CRI 1.22 to 2.95, Fig 2C).

### Ovenbirds

From 2012–2015 we monitored 94 ovenbird nests and attached radios to 62 ovenbirds from 48 broods. We relocated ovenbirds every 1.48 ± 1.01 days for a maximum of 49 days [18,32]. We collected 53 complete choice-sets for ovenbird nests and 508 choice-sets for 42 ovenbird juveniles with 12.1 ± 1.16 (range 1−27) sets per individual (163 sets from 42 early dependent individuals, 244 choice-sets from 29 late dependent individuals, and 102 choice-sets from 28 independent individuals) from 2012–2015 (S3 Table). The ovenbird postfledging model would not converge when coefficients were modeled separately for the independent period, suggesting that our data could not support non-random selection after independence. The final converging ovenbird postfledging model included population-level coefficients modeled separately for choice-sets sampled within one week of fledging (early dependent), and for fledglings > 8 days post-fledge (late dependent & independent). Both nest and postfledging models performed better than a null model of random selection; Estrella’s R^2^ indicated ovenbird nest and postfledging selection models had moderate predictive accuracy overall, 0.30 and 0.22, respectively.

**Fig 3:**
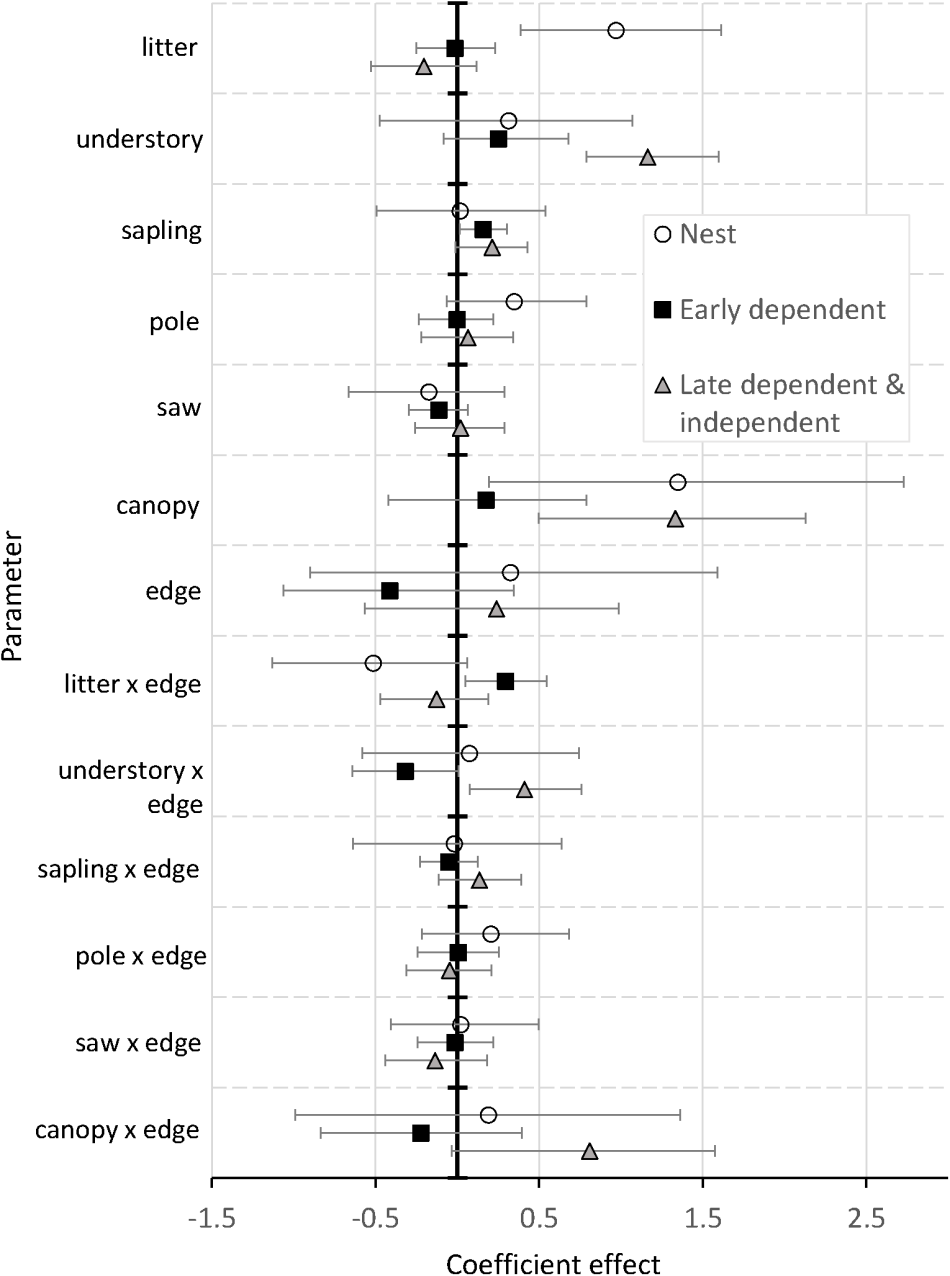
Parameter coefficients from ovenbird discrete choice models. Mean population-level parameter coefficients and 95% credible intervals (bars) from ovenbird discrete choice models for nest sites, dependent fledglings in the first week out of the nest (early dependent), and fledglings after the first week out of the nest (late dependent and independent) in Missouri from 2012−2015.

**Fig 4:**
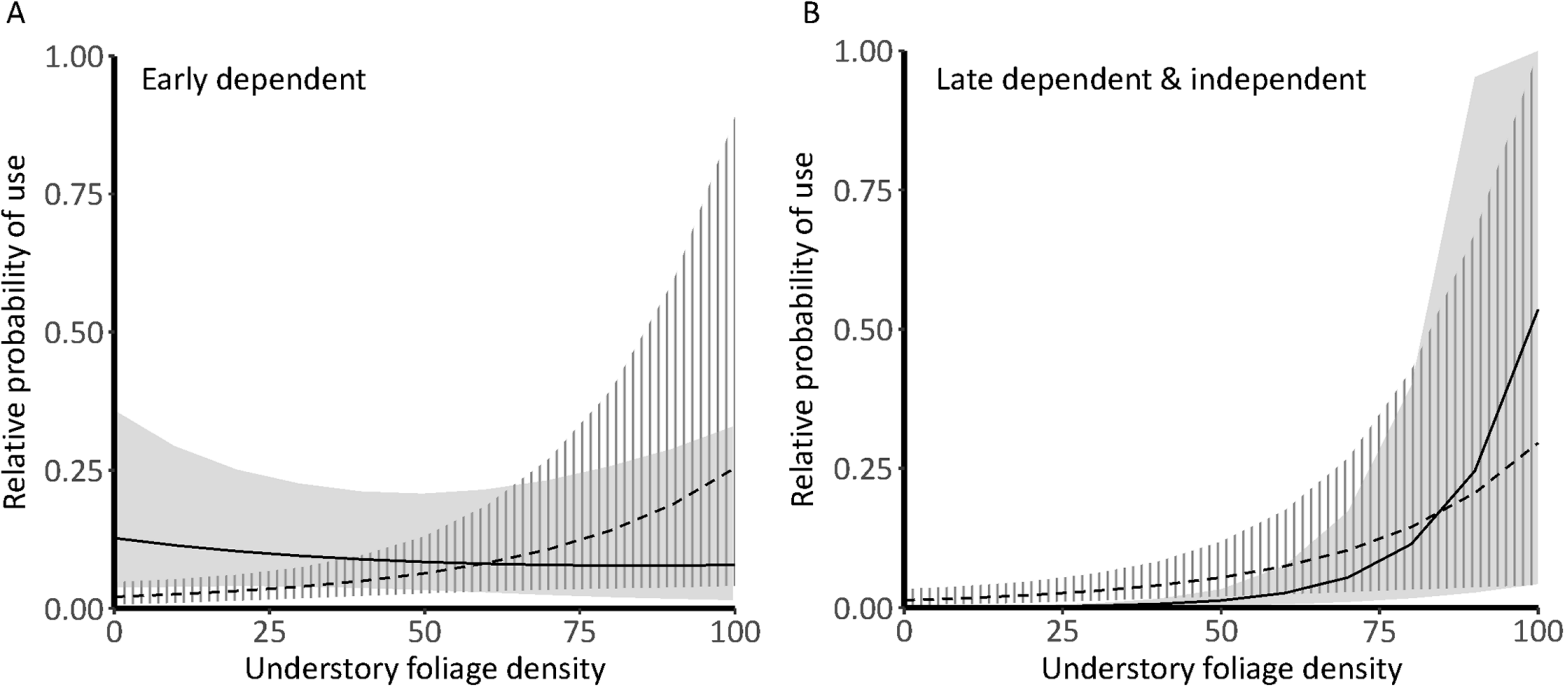
Understory foliage density affected location utility for postfledging ovenbirds. Estimated relative probability of use (±95% credible intervals) as a function of understory foliage density for ovenbirds in the first 7 days postfledging (A) and after 7 days postfledging (B) for birds near (dotted lines) and far (solid lines) from forest edge in Missouri, 2012−2015. Probability curves were created by holding non-focal covariates at their means and distance to edge at the 10^th^ percentile (near edge = 10 m) and 90^th^ percentile (far from edge = 300 m).

Coefficient CRIs did not overlap across the three breeding season stages for litter depth, understory foliage density, and the interaction of edge and understory foliage density, suggesting changes in their utility among stages (Fig 3, S4 Table). Litter depth positively affected nest site selection (*f* = 1) and interacted with distance to edge (*f* = 0.96). Litter depth had a neutral effect on early dependent resource selection (*f* = 0.55) and had a negative association with late dependent and independent postfledging selection (*f* = 0.89) with a selection ratio of 0.82 (95% CRI 0.59 to 1.13). Sapling density, which was not associated with nest site selection (*f* = 0.54), was positively associated with both periods of postfledging selection (*f* ≥ 0.96, Fig 3). The effect of understory foliage density on nest site selection had a mean selection ratio of 1.37 but was widely distributed (95% CRI 0.62 to 2.91), suggesting high variability in utility population-wide. Understory density was positively associated with selection (*f* ≥ 0.92) and interacted with distance to edge in both postfledging stages (*f* ≥ 0.97, Fig 3, Fig 4). The mean effect of understory foliage was 4.6x greater after the first week out of the nest (β_Early_ = 0.25, β_Late_ = 1.16) and interaction coefficients flipped from the early postfledging period to the late dependent and independent period (β_Early_ = -0.32, β_Late_ = 0.41). Selection by early postfledging individuals near a nonforest edge was less associated with understory foliage density, compared to individuals far from edge areas (Fig 4A). Late dependent individuals were more likely to select areas with higher understory density regardless of distance to edge (Fig 4B).

## Discussion

Vegetation characteristics selected changed over the course of the breeding season for both ovenbirds and Acadian flycatchers, suggesting that the relative importance of certain habitat characteristics changed with juvenile development. The timing and extent of shifting resource selection was species-specific. Acadian flycatchers altered selection only after independence from adults, while ovenbirds altered selection immediately after fledging. We suggest that changes in resource selection requirements may be linked to foraging method, prey distribution, and juvenile mobility at fledging. Acadian flycatchers were able to glide or make short flights in the first week, while ovenbirds were unable to fly for 3−4 days postfledging [18]. In general, there was not a great deal of heterogeneity within choice-sets from our mature forest areas (S1 and S3 Tables); low heterogeneity within choice-sets likely made our utility estimates conservative, weakening our ability to detect the utility for some variables of interest [40]. Since we constrained sampling largely to the morning, our findings are representative of changing postfledging foraging site selection only; roost site selection may be equally important and could follow different patterns [41].

Our results for ovenbird nest site selection are consistent with previous findings that ovenbirds select nest sites with high canopy cover, mature forest, low ground cover, and high litter depths [14]. We were not able to model late dependent and independent postfledging resource selection separately for ovenbirds. The lack of model convergence for population-level independent juvenile selection was likely due to reduced variability between our sampled choice-sets in that category (S3 Table); independent juveniles tended to move to larger patches of relatively homogenous dense vegetation. We did not observe any evidence in the field that would suggest much difference in selection between late dependent juveniles and independent juveniles. Studies in Missouri, Minnesota and Ohio also reported independent juvenile ovenbirds spending time in a variety of cover types with dense understory vegetation [7,21,42].

The similarity between nesting and dependent-postfledging resource selection by Acadian flycatchers is not surprising given that open areas under closed canopy are required for both flycatcher nesting (mobility) and foraging [13]. This is consistent with prior studies that found that Acadian flycatchers place nests more often in areas with open understory [13,43]. Postfledging flycatchers in our study appeared less restricted than nesting birds in their vertical space-use, utilizing all canopy layers, whereas nesting flycatchers were mainly observed foraging and defending nests in the low or mid-canopy [18]. We may have missed an important structural requirement for postfledging flycatcher resource selection, since our traditional plot sampling was not designed to capture comprehensive vertical canopy structure. Future research for species that forage by sallying would benefit from measurements designed to capture vertical forest structure in greater depth. The change in flycatcher resource selection post-independence may be driven by an increase in vigilance after juveniles leave adult care, or this shift could be due to competitive exclusion from habitats preferred by adults.

Increased forest edge and forest fragmentation are often hypothesized to increase risk for many forest songbird species because they can increase activity or abundance of predators such as snakes, raccoons, and jays [44]. We did not detect strong avoidance of edge by either species, as observed in some studies [45,46], although this may be due to the relatively fine scale at which we sampled random available locations (~50 m). We detected changes in the utility of understory foliage density depending on the choice-set’s distance to edge in both species (Fig 2, Fig 4), which may reflect a difference in perceived predator communities near and far from edges. Alternatively, since there are generally higher densities of vertical cover near edge, cover from predators may become less important than open foraging space for sally foragers, like the Acadian flycatcher.

Ultimate factors for habitat selection include the ability of a chosen location to provide protection from the elements or from predators and to provide food [47]. The primary cause of juvenile mortality for forest breeding songbirds in both the nest and postfledging stages is predation [5,44], suggesting that protection from predators is key for these forest breeding species throughout the breeding season. Nest site selection likely evolved to maximize protection of a single stationary location [48], from which adults can visit with minimal detection by predators. Postfledging diurnal resource selection likely evolved to maximize protection from predation while foraging. Species-specific variation then should be common due to variation across species’ life histories. While the use of thick vegetation has been reported for fledglings of several forest and grassland nesting birds, including: northern cardinals (*Cardinalis cardinalis* [17]), dickcissels (*Spiza americana* [49]), hooded warblers (*Setophaga citrina* [50]), Swainson’s thrush (*Catharus ustulatus* [51]), wood thrush (*Hylocichla mustelina* [9]), and worm-eating warblers (*Helmitheros vermivorum* [42]), the extent of use and the timing of shifts in selection to dense cover are not clear or uniformly expressed. Burke (2013) followed independent red-eyed vireos (*Vireo olivaceus*), worm-eating warblers, and ovenbirds captured in regenerating clearcut stands and found that while all three forest nesting species utilized the dense regenerating forest for foraging, red-eyed vireos spent an equal proportion of time outside of the dense vegetation in mature forest areas [7]. So, while the ultimate cause of resource selection postfledging may be to avoid predators while collecting food, differences in foraging strategy, mobility, and competitive interactions likely shape observed habitat selection patterns across the breeding season for individual species.

Breeding density and productivity are important elements for understanding breeding ecology of migrant songbirds. However, if a species is abundant and successful at producing young, but those young do not survive through the summer to migration, there is no benefit to the population. Given that there are high rates of mortality in many postfledging birds, we need to consider both nesting and postfledging habitat for population management and conservation efforts to be successful [4]. In our study, Ovenbird resource selection changed during the period of highest postfledge mortality, while Acadian flycatcher selection did not [32]. We illustrate that species which require similar nesting habitat can have meaningfully different patterns of resource selection over the course of the breeding season (nesting through postfledging). Variation in the timing and extent of changes in resource use across the breeding season is likely common in migratory songbirds, however, information on the postfledging period is lacking for the majority of species [5]. This variation may have strong implications for resource managers when considering the needs of birds that change their habitat use over the course of the breeding season. We suggest species-specific conservation efforts that address multiple stages of avian life history, particularly focusing on stages where mortality rates are high, may be necessary for effective conservation of migrant songbirds.

## Supporting information

S1 Dataset Relevant data used in discrete choice analysis. (XLSX)

S1 Table Acadian flycatcher selection variables. Arithmetic mean values, standard errors (SE), and sample size (n) from vegetation sampled at used and random locations for nests, early dependent fledglings, late dependent fledglings, and independent Acadian flycatchers in Missouri from 2013–2015.
(DOCX)

S2 Table Covariate effects for Acadian flycatcher discrete choice models. Posterior coefficient distribution means (β) and the proportion of the posterior with the same sign as the mean (*f*) from models of Acadian flycatcher resource selection for nest sites, early dependent fledglings, late dependent fledglings, and independent fledglings in Missouri from 2013–2015.
(DOCX)

S3 Table Ovenbird selection variables. Arithmetic mean values, standard errors (SE), and sample size (n) from vegetation sampled at used and random locations for nests, early dependent fledglings, and late dependent and independent fledgling ovenbirds in Missouri from 2012–2015.
(DOCX)

S4 Table Covariate effects for Ovenbird discrete choice models. Posterior coefficient distribution means (β) and the proportion of the posterior with the same sign as the mean (*f*) from models of Ovenbird resource selection for nest sites, early dependent fledglings, and late dependent and independent fledglings in Missouri from 2012–2015.
(DOCX)
