# Supplementary material for: Species-specific variation in nesting and postfledging resource selection for two forest breeding migrant songbirds
Source: PLoS One. 2017 Jun 14;12(6):e0179524. doi: 10.1371/journal.pone.0179524 (PMC5470712; doi:10.1371/journal.pone.0179524)
Supplement: S1 Table — Arithmetic mean values, standard errors (SE), and sample size (n) from vegetation sampled at used and random locations for nests, early dependent fledglings, late dependent fledglings, and independent Acadian flycatchers in Missouri from 2013–2015. (DOCX) [file pone.0179524.s002.docx]

|  | | Acadian Flycatcher use | | | |  | Acadian Flycatcher random | | | |
| --- | --- | --- | --- | --- | --- | --- | --- | --- | --- | --- |
|  | | Mean | ± | SE | n |  | Mean | ± | SE | n |
| Nest | |  |  |  |  |  |  |  |  |  |
|  | Litter depth | 1.638 | ± | 0.090 | 170 |  | 1.707 | ± | 0.059 | 340 |
|  | Foliage density (0-2m) | 42.510 | ± | 2.886 | 170 |  | 46.606 | ± | 2.251 | 340 |
|  | Saplings (stems/ha) | 151.328 | ± | 11.541 | 170 |  | 174.715 | ± | 10.114 | 340 |
|  | Pole timber (stems/ha) | 694.118 | ± | 62.852 | 170 |  | 645.145 | ± | 48.794 | 340 |
|  | Saw timber (stems/ha) | 99.982 | ± | 4.871 | 170 |  | 104.972 | ± | 2.959 | 340 |
|  | Canopy cover | 95.436 | ± | 0.293 | 170 |  | 94.223 | ± | 0.357 | 340 |
|  | Distance to edge | 142.795 | ± | 7.927 | 170 |  | 142.265 | ± | 5.672 | 340 |
| Early dependent | |  |  |  |  |  |  |  |  |  |
|  | Litter depth | 1.831 | ± | 0.097 | 138 |  | 1.765 | ± | 0.062 | 276 |
|  | Foliage density (0-2m) | 31.051 | ± | 2.804 | 138 |  | 35.317 | ± | 2.119 | 276 |
|  | Saplings (stems/ha) | 125.279 | ± | 12.293 | 138 |  | 159.003 | ± | 8.914 | 276 |
|  | Pole timber (stems/ha) | 779.614 | ± | 85.262 | 138 |  | 675.748 | ± | 53.195 | 276 |
|  | Saw timber (stems/ha) | 123.626 | ± | 5.043 | 138 |  | 131.525 | ± | 4.448 | 276 |
|  | Canopy cover | 96.187 | ± | 0.243 | 138 |  | 95.091 | ± | 0.256 | 276 |
|  | Distance to edge | 144.040 | ± | 9.886 | 138 |  | 144.475 | ± | 6.830 | 276 |
| Late dependent | |  |  |  |  |  |  |  |  |  |
|  | Litter depth | 1.950 | ± | 0.091 | 172 |  | 1.738 | ± | 0.059 | 344 |
|  | Foliage density (0-2m) | 39.855 | ± | 2.903 | 172 |  | 38.302 | ± | 1.853 | 344 |
|  | Saplings (stems/ha) | 209.118 | ± | 15.496 | 172 |  | 187.004 | ± | 9.469 | 344 |
|  | Pole timber (stems/ha) | 742.799 | ± | 61.544 | 172 |  | 676.386 | ± | 44.090 | 344 |
|  | Saw timber (stems/ha) | 114.730 | ± | 4.904 | 172 |  | 120.461 | ± | 3.532 | 344 |
|  | Canopy cover | 95.639 | ± | 0.288 | 172 |  | 95.511 | ± | 0.215 | 344 |
|  | Distance to edge | 142.388 | ± | 8.576 | 172 |  | 140.058 | ± | 6.024 | 344 |
| Independent | |  |  |  |  |  |  |  |  |  |
|  | Litter depth | 1.522 | ± | 0.094 | 112 |  | 1.562 | ± | 0.071 | 224 |
|  | Foliage density (0-2m) | 40.321 | ± | 3.744 | 112 |  | 40.695 | ± | 2.851 | 224 |
|  | Saplings (stems/ha) | 238.224 | ± | 22.395 | 112 |  | 212.305 | ± | 14.215 | 224 |
|  | Pole timber (stems/ha) | 858.476 | ± | 91.038 | 112 |  | 888.547 | ± | 62.944 | 224 |
|  | Saw timber (stems/ha) | 106.317 | ± | 6.694 | 112 |  | 107.832 | ± | 4.496 | 224 |
|  | Canopy cover | 95.582 | ± | 0.623 | 112 |  | 93.426 | ± | 0.772 | 224 |
|  | Distance to edge | 132.957 | ± | 10.214 | 112 |  | 131.344 | ± | 7.229 | 224 |
