# Supplementary material for: Species-specific variation in nesting and postfledging resource selection for two forest breeding migrant songbirds
Source: PLoS One. 2017 Jun 14;12(6):e0179524. doi: 10.1371/journal.pone.0179524 (PMC5470712; doi:10.1371/journal.pone.0179524)
Supplement: S2 Table — Posterior coefficient distribution means (β) and the proportion of the posterior with the same sign as the mean (f) from models of Acadian flycatcher resource selection for nest sites, early dependent fledglings, late dependent fledglings, and independent fledglings in Missouri from 2013–2015. (DOCX) [file pone.0179524.s003.docx]

|  | Nest | |  | Early dependent | |  | Late dependent | |  | Independent | |
| --- | --- | --- | --- | --- | --- | --- | --- | --- | --- | --- | --- |
| Covariate | β | *f* |  | β | *f* |  | β | *f* |  | β | *f* |
| Litter depth | -0.03 | 0.56 |  | 0.20 | 0.92 |  | -0.11 | 0.73 |  | 0.66 | 1.00 |
| Understory foliage density | -0.24 | 0.92 |  | -0.06 | 0.64 |  | -0.46 | 0.99 |  | 0.63 | 1.00 |
| Saplings | 0.04 | 0.67 |  | 0.10 | 0.82 |  | -0.06 | 0.66 |  | -0.05 | 0.64 |
| Pole timber | -0.28 | 0.98 |  | -0.12 | 0.80 |  | 0.19 | 0.87 |  | -0.23 | 0.91 |
| Saw timber | -0.23 | 0.97 |  | -0.32 | 0.99 |  | -0.04 | 0.60 |  | -0.13 | 0.73 |
| Canopy cover | 0.79 | 1.00 |  | 0.32 | 0.89 |  | 0.62 | 0.97 |  | 0.35 | 0.92 |
| Distance to edge | -0.02 | 0.53 |  | -0.30 | 0.74 |  | 0.55 | 0.83 |  | 1.43 | 1.00 |
| litter × edge | 0.54 | 1.00 |  | 0.16 | 0.89 |  | -0.07 | 0.67 |  | 0.10 | 0.71 |
| under × edge | -0.04 | 0.56 |  | -0.04 | 0.59 |  | 0.52 | 0.98 |  | 0.00 | 0.51 |
| sapling × edge | -0.01 | 0.55 |  | -0.16 | 0.89 |  | -0.22 | 0.90 |  | -0.10 | 0.74 |
| pole × edge | 0.13 | 0.81 |  | -0.18 | 0.89 |  | -0.03 | 0.58 |  | 0.02 | 0.54 |
| saw × edge | -0.13 | 0.81 |  | -0.04 | 0.61 |  | 0.08 | 0.65 |  | -0.04 | 0.59 |
| canopy × edge | 0.25 | 0.89 |  | -0.09 | 0.66 |  | -0.02 | 0.52 |  | 0.19 | 0.81 |
