# Supplementary material for: Species-specific variation in nesting and postfledging resource selection for two forest breeding migrant songbirds
Source: PLoS One. 2017 Jun 14;12(6):e0179524. doi: 10.1371/journal.pone.0179524 (PMC5470712; doi:10.1371/journal.pone.0179524)
Supplement: S3 Table — Arithmetic mean values, standard errors (SE), and sample size (n) from vegetation sampled at used and random locations for nests, early dependent fledglings, and late dependent and independent fledgling ovenbirds in Missouri from 2012–2015. (DOCX) [file pone.0179524.s004.docx]

|  | | Ovenbird use | | | |  | Ovenbird random | | | |
| --- | --- | --- | --- | --- | --- | --- | --- | --- | --- | --- |
|  | | Mean | ± | SE | n |  | Mean | ± | SE | n |
| Nest | |  |  |  |  |  |  |  |  |  |
|  | Litter depth | 2.546 | ± | 0.149 | 53 |  | 2.088 | ± | 0.112 | 106 |
|  | Foliage density (0-2m) | 60.509 | ± | 5.433 | 53 |  | 57.987 | ± | 3.943 | 106 |
|  | Saplings (stems/ha) | 149.121 | ± | 19.500 | 53 |  | 118.979 | ± | 12.807 | 106 |
|  | Pole timber (stems/ha) | 644.047 | ± | 114.570 | 53 |  | 600.338 | ± | 82.352 | 106 |
|  | Saw timber (stems/ha) | 105.088 | ± | 7.282 | 53 |  | 109.632 | ± | 5.411 | 106 |
|  | Canopy cover | 94.074 | ± | 0.562 | 53 |  | 92.367 | ± | 0.893 | 106 |
|  | Distance to edge | 174.976 | ± | 13.955 | 53 |  | 171.377 | ± | 9.703 | 106 |
| Early dependent | |  |  |  |  |  |  |  |  |  |
|  | Litter depth | 2.070 | ± | 0.099 | 163 |  | 2.164 | ± | 0.080 | 324 |
|  | Foliage density (0-2m) | 64.171 | ± | 3.584 | 163 |  | 59.552 | ± | 2.670 | 324 |
|  | Saplings (stems/ha) | 185.675 | ± | 18.517 | 163 |  | 194.065 | ± | 12.771 | 324 |
|  | Pole timber (stems/ha) | 750.443 | ± | 76.807 | 163 |  | 569.305 | ± | 44.212 | 324 |
|  | Saw timber (stems/ha) | 118.391 | ± | 5.658 | 163 |  | 116.375 | ± | 3.407 | 324 |
|  | Canopy cover | 94.682 | ± | 0.522 | 163 |  | 92.811 | ± | 0.730 | 324 |
|  | Distance to edge | 151.515 | ± | 9.131 | 163 |  | 149.102 | ± | 6.213 | 324 |
| Late dependent & independent | |  |  |  |  |  |  |  |  |  |
|  | Litter depth | 1.914 | ± | 0.073 | 345 |  | 1.939 | ± | 0.052 | 692 |
|  | Foliage density (0-2m) | 74.702 | ± | 2.954 | 345 |  | 62.600 | ± | 1.907 | 692 |
|  | Saplings (stems/ha) | 222.314 | ± | 11.911 | 345 |  | 206.150 | ± | 7.789 | 692 |
|  | Pole timber (stems/ha) | 757.283 | ± | 47.746 | 345 |  | 612.992 | ± | 30.228 | 692 |
|  | Saw timber (stems/ha) | 99.107 | ± | 3.503 | 345 |  | 104.770 | ± | 2.400 | 692 |
|  | Canopy cover | 92.569 | ± | 0.619 | 345 |  | 88.237 | ± | 0.849 | 692 |
|  | Distance to edge | 114.650 | ± | 6.018 | 345 |  | 116.912 | ± | 4.170 | 692 |
